# Evaluation of the Efficacy and Safety of Anifrolumab in Moderate-to-Severe Systemic Lupus Erythematosus

**DOI:** 10.7759/cureus.63966

**Published:** 2024-07-06

**Authors:** Ananya Reddy Cingireddy, Navya Ramini, Anirudh Reddy Cingireddy

**Affiliations:** 1 Internal Medicine, Mountain View Regional Medical Center, Las Cruces, USA; 2 Anesthesiology and Critical Care, All India Institute of Medical Sciences, Raipur, IND; 3 Computer Science, East Central University, Ada, USA

**Keywords:** safety, efficacy, interferon signature, systemic lupus erythematosus, anifrolumab

## Abstract

Systemic lupus erythematosus (SLE) is an autoimmune disease, which poses significant challenges due to its chronic nature and complex clinical manifestations. For patients with moderate-to-severe SLE, anifrolumab, a monoclonal antibody that targets the type 1 interferon receptor (IFNAR), has emerged as a cutting-edge treatment option that can reduce disease activity, prevent organ damage from the illness or side effects resulting from medications, and enhance the quality of life for those living with SLE. Consequently, this drug has received approval from major regulatory agencies. Anifrolumab's safety, effectiveness, and long-term results are assessed in this systematic review using information from clinical trials, real-world research, and retrospective analysis. In particular, clinical investigations, such as the MUSE Phase II and TULIP Phase III trials, showed that anifrolumab significantly improved important outcomes compared to placebo, including the SLE Responder Index, major clinical response, and disease activity ratings. During extended use, anifrolumab demonstrated significant sustained efficacy and a tolerable safety profile, with controllable side events mostly associated with viral infections. Moreover, subgroup analyses, demonstrating that Asian patients and individuals with a strong interferon gene profile are particularly responsive to anifrolumab, underscore the importance of customized treatment methods. Anifrolumab’s safety and effectiveness were further validated by real-world data, particularly in patients who reached the Lupus Low Disease Activity State (LLDAS), where the drug decreased glucocorticoid consumption and disease activity. Overall, anifrolumab shows great promise as a treatment for moderate-to-severe SLE, providing significant efficacy together with a manageable safety profile. To fully explore its therapeutic potential and optimize therapy approaches for the management of SLE, further research is necessary, especially in lupus nephritis and other disease subsets.

## Introduction and background

Systemic lupus erythematosus (SLE) is the most common type of lupus and is defined as a chronic autoimmune condition that affects multiple systems and is characterized by relapsing and remitting episodes [1]. The immunopathogenesis of SLE is characterized by elevated levels of circulating cell death debris, resulting from impaired clearance of apoptotic cells and neutrophil extracellular traps (NETs). This accumulation of self-antigens leads to heightened activation of the type I interferon (IFN-I) pathway and the formation of immune complexes, driving an inflammatory response that contributes to tissue damage [2]. It has been shown that women of reproductive age are more subjected to developing SLE, with a female predominance of 9:1 [3]. The clinical spectrum of this illness encompasses a broad range of symptoms, spanning from fever and malaise to joint pain and weight changes, alongside varying degrees of cutaneous involvement and extending to severe organ damage such as heart failure, pulmonary hypertension, and kidney failure [4-6]. Often symptoms are nonspecific and can mimic other conditions such as autoimmune diseases, infections, and endocrine abnormalities [6]. 

Clinical manifestations of SLE

In patients affected by SLE, musculoskeletal involvement is quite common, with joint pain being a primary reason for seeking medical attention [7]. Arthritis, arthralgia, and osteonecrosis are prevalent, affecting small joints asymmetrically and typically without significant deformity [6]. Cutaneous manifestations are characterized by four main hallmarks, those being malar rash, photosensitivity, discoid rash, and alopecia [6]. Renal involvement is also quite common in patients with SLE, with immunoglobulin deposits in glomeruli often present despite only half of the patients exhibiting clinical symptoms [8]. Additionally, neuropsychiatric symptoms such as cognitive disorders, seizures, and neuropathies, along with pulmonary manifestations such as pleurisy and pulmonary embolism, may arise as clinical manifestations of SLE [6,8]. Other common features are gastrointestinal involvement, which often presents with abdominal pain and nausea, and cardiac manifestations ranging from pericarditis to accelerated coronary artery disease [6,9]. Vascular complications, including Raynaud's phenomenon and vasculitis as well as ocular manifestations, may also occur [6]. Furthermore, hematologic abnormalities, such as cytopenias and thrombocytopenia, are common in SLE patients and are often accompanied by elevated inflammatory markers [6]. Also, flares can happen frequently, and remission can be hard to induce and sustain [10]. Researchers and clinicians in the field are still actively studying the factors underlying lupus causes and symptoms. The most comprehensive and accurate classification criteria to date are those employed by the American College of Rheumatology (ACR) and the European League Against Rheumatism (EULAR) [11].

Genetic and environmental influences in SLE

Although the precise pathophysiology of SLE remains unclear, patients present with vasculopathy, deposition of immunological complexes (ICs) in multiple organs, and an overall inflammatory environment [1]. The clinical heterogeneity of SLE revealed that the disease pathology is influenced by a variety of susceptibility variables, including genetic, epigenetic, environmental, infectious, and hormonal factors [1]. The development of SLE is mainly caused by a number of genes, the interaction of sex hormones, and faulty immune regulatory systems, including poor removal of apoptotic cell debris and immune complex deposition [5,12]. Genome-wide association studies (GWAS) have identified over 100 gene loci associated with SLE susceptibility, categorized into four main groups [13-15]. The first group includes genes involved in apoptosis, autophagy, DNA repair, lysosome function, and immune complex clearance [14]. The second group encompasses genes related to innate immunity and associated signaling pathways like IFN-I, toll-like receptors (TLR), and nuclear factor κB (NFκB) [15]. Approximately 50% of SLE patients have chronically high blood levels of IFN-I [16]. Also, the "IFN signature," characterized by the overexpression of IFN-I pathway genes in peripheral blood cells, has been observed in an even higher proportion of individuals [17-19]. IFN signature genes can also be induced by other pathways and downstream effectors [20-22]. Elevated IFN-1 activity has been correlated with other cytokines, such as B-cell activating factor (BAFF) and type II IFN (IFN-II), along with certain autoantibodies and clinical symptoms like lupus nephritis [23-25]. The third group consists of genes involved in adaptive immunity, including HLA and non-HLA genes [15]. The fourth group comprises genes with known immune functions but unclear roles in SLE [15]. These genetic factors explain about 30-50% of SLE heritability, with other contributions from rare genetic variants, epigenetic changes, and gene interactions [26,27]. On this account, numerous epigenetic alterations, including methylation, acetylation, and short RNA, have also been discovered to influence the pathophysiology of the disease; yet, as these modifications might differ from one another, a customized strategy is needed to clarify the function of these mechanisms in lupus patients [10].

Corticosteroids and immunosuppressant medications are commonly used in combination as current treatments for SLE; however, many of these medications have serious adverse effects and are not FDA-approved for the condition [28]. Despite advances in treatment, individuals with moderate-to-severe SLE still face a large illness burden and few therapeutic options. In over 50 years, the FDA has only approved two novel molecularly targeted medications as adjuvant therapy for adult SLE patients namely: belimumab and anifrolumab, which recognize BAFF and the IFN-I receptor, respectively [29,30].

Anifrolumab treatment in SLE

The pivotal role of IFN-I in SLE pathogenesis led to the development of anifrolumab (Saphnelo™), a human IgG1κ monoclonal antibody that antagonizes the type 1 interferon receptor subunit 1 (IFNAR1) to inhibit IFN-I signaling, involved in innate and adaptive antiviral immunity across various cells [2,30]. By doing so, anifrolumab impedes the formation of the IFN/IFNAR complex, consequently inhibiting subsequent gene transcription. Also, by targeting the receptor involved in the cellular signaling, anifrolumab neutralizes several interferons including IFN-α, IFN-β, IFN-ε, IFN-κ, and IFN-ω [31]. This inhibition prevents the activation of the IFN-I pathway, which is known to play a central role in the immunopathogenesis of SLE and thus helps correct the dysregulated immune responses seen in SLE.

Anifrolumab has been approved for the treatment of adult patients with moderate-to-severe SLE based on findings from the phase 2 MUSE trial and two phase 3 trials, TULIP-1 and TULIP-2 [2]. It was developed by AstraZeneca for the treatment of autoimmune disorders, including SLE and lupus nephritis, whose underlying pathogenesis involves IFN-I [30]. Before being approved by major regulatory agencies, in 2021 by the FDA and 2022 by the EMA, anifrolumab underwent several clinical trials including the two TULIP Phase III trials and the MUSE Phase II trial [32,33]. In particular, analysis of data obtained from the trials indicated that anifrolumab suppressed inflammatory proteins associated with disease activity, improved markers of cardiometabolic disease, and reversed SLE-related cytopenias, supporting the broad impact of IFNAR1 blockade [2].

The aim of this systematic review was to compile and evaluate the available data on anifrolumab's safety, efficacy, and long-term results for treating moderate-to-severe SLE, taking into account information from clinical trials as well as empirical data. To fully understand anifrolumab's clinical utility, it is essential not only to summarize data from clinical trials but also to explore the subtleties of the drug's performance in different patient subgroups. Through the assessment of anifrolumab's safety, efficacy, and pharmacokinetics in a range of demographic groups and disease phenotypes, this review aims to provide a more comprehensive understanding of the drug's potential for therapeutic use in real-world clinical scenarios. Hopefully, through the identification of patient variables that may impact therapy outcomes, this study will aid in the progress toward more personalized therapeutic techniques to meet the needs of each particular patient.

Research question

The research question of this systematic review was to evaluate the safety, efficacy, and long-term effectiveness of anifrolumab for treating moderate-to-severe SLE, based on available data from clinical trials and empirical research.

Research objective

The objective of this systematic review was to evaluate all available data, including real-world studies and clinical trial data, regarding the use of anifrolumab in the treatment of moderate-to-severe SLE. We aimed to offer insights into the drug's therapeutic potential and support clinical decision-making by synthesizing this evidence.

Research rationale

The rationale for conducting this study stems from the need to address the unmet medical need in SLE management and explore innovative treatment approaches. Anifrolumab has emerged as a promising therapy for SLE, offering the potential to reduce disease activity, prevent organ damage, and improve patient’s quality of life. 

## Review

Methodology 

Search Strategy 

A systematic search was conducted in PubMed using the following keywords: "anifrolumab," "systemic lupus erythematosus," "efficacy," and "safety." The search was limited to articles published 1st of May 2024.

The inclusion criteria for this systematic review include publications that assess anifrolumab's safety and effectiveness in treating SLE, that were published by the specified date, that involved human subjects, and that reported original research findings, including observational studies, case-control studies, and clinical trials. On the other hand, the exclusion criteria comprise medical letters and documentation that do not provide new information regarding anifrolumab's safety and efficacy in the treatment of SLE, systematic reviews and meta-analyses comparing different biological agents in SLE that are considered outdated, and articles that concentrate on lupus nephritis rather than SLE. 

Article Selection 

Initially, 40 articles were identified through the search. Six articles that addressed lupus nephritis rather than SLE were disqualified from consideration. Moreover, three papers were eliminated because they compared various biological agents in SLE through systematic reviews and meta-analyses, which were judged out of date given the field's developments. Finally, four medical letters and documentation were not included in the evaluation as they did not provide newer findings regarding the efficacy and safety of the drug. All 27 remaining articles identified during the initial search process were utilized for this systematic review. In addition to the articles retrieved from the initial search, an extra article was included based on its relevance to the topic under investigation.

Data Items Extraction and PICO Framework

From the 28 remaining articles selected for screening, a meticulous extraction process was undertaken to collect relevant data about the study's methodology. This included information about the study's design (e.g., observational studies, randomized controlled trials (RCT), or case-control studies), as well as specific data items according to the PICO framework, which stands for population, intervention, comparison, and outcome.

Population: The study participants' demographic attributes, including their ethnicity, as well as information about the disease's attributes, such as its duration and severity, were documented and reported in the chosen publications. 

Intervention: Detailed information about anifrolumab administration was recorded, including dosage, frequency, duration, and mode of administration.

Comparison*:* In the included studies, anifrolumab was compared to both placebo and standard of treatment allowing us to evaluate its safety and efficacy, as well as how well it worked in comparison to other established treatments for SLE.

Outcome: Detailed summaries of the research were collected, covering efficacy outcomes such as clinical response rates, disease activity metrics, and changes in biomarkers. All adverse events (AEs) and safety concerns pertaining to the administration of anifrolumab were also recorded.

Table 1 outlines the details of the systematic search conducted in PubMed for articles related to anifrolumab's safety and efficacy in treating SLE.

**Table 1:** Summary of the specifics of the systematic search for publications about the safety and effectiveness of anifrolumab in treating SLE.

Search number	Query	Filters	Search details	Results
1	"anifrolumab" AND "systemic lupus erythematosus" AND "efficacy" AND "safety"	- Publication Date: Up to 1st May 2024 - Article type: Original research - Study types: Observational studies, case-control studies, clinical trials	Conducted a systematic search in PubMed using the mentioned keywords with a publication date restriction up to 1st May 2024. Excluded articles that did not meet inclusion criteria.	40 articles were identified, and 27 were retained after exclusions. 1 extra article was included based on relevance to the topic.

*PRISMA Guidelines* 

**Figure 1:**
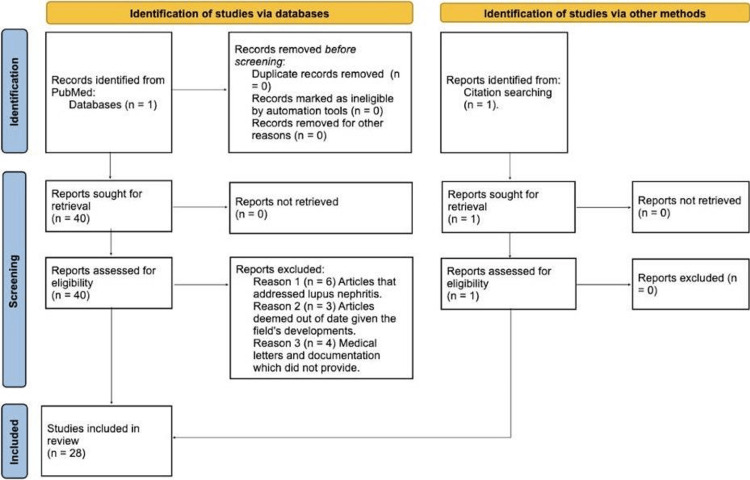
PRISMA chart representing the systematic process employed for selecting studies to be included in the systematic review on the safety and efficacy of anifrolumab in treating SLE. PRISMA, Preferred Reporting Items for Systematic Reviews and Meta-Analyses

To ensure transparency and adherence to rigorous systematic review methods, the Preferred Reporting Items for Systematic Reviews and Meta-Analyses (PRISMA) guidelines was followed. Figure 1 shows a PRISMA flowchart that describes the study selection procedure. This flowchart presents the totality of articles selected, screened, and included at each stage as well as the rationale behind their exclusion, along with the methodical search approach that was used.

*Quality Assessment* 

Ensuring that only studies of high methodological quality were included in the review, this assessment covered important factors like study design appropriateness, sample size adequacy, implementation of blinding techniques (i.e., single-blind, double-blind), proper randomization procedures, allocation concealment methods (i.e., randomization) to minimize bias and robust strategies for handling missing data.

*Data Interpretation* 

An extensive analysis was conducted to synthesize and evaluate the retrieved data in order to get valuable insights into the safety and efficacy of anifrolumab as a treatment for SLE. In order to reach well-founded conclusions about the drug's therapeutic potential in managing SLE, this process involved carefully weighing the advantages and disadvantages of the included studies, spotting trends among disparate research findings, and critically evaluating the body of available data. The interpretation phase also involved addressing any discrepancies or conflicting results among the included studies, ensuring a comprehensive and nuanced understanding of anifrolumab's role in SLE management.

Results

Evaluating Safety, Effectiveness, and Long-Term Results of Anifrolumab in Clinical Trials of Moderate-to-Severe SLE

Before being approved by major regulatory agencies, anifrolumab underwent several clinical trials including two TULIP Phase III trials and the MUSE Phase II trial [32,33]. 

During the MUSE Phase IIb (NCT01438489), a randomized, double-blind, placebo-controlled study, anifrolumab was assessed for efficacy and safety in adults with moderate-to-severe SLE [34]. For 48 weeks, patients (n=305) received conventional therapy in addition to intravenous anifrolumab (300 mg or 1000 mg) or placebo every four weeks. A considerably higher percentage of patients treated with anifrolumab than with placebo achieved the primary endpoint, the SLE Responder Index SRI (4) response at week 24, which included a sustained reduction of oral corticosteroids, improvement of skin disease, and a reduction of flare rates [34]. In patients with a strong baseline IFN signature, the effect was more noticeable. Monthly intravenous administration of 300 mg of anifrolumab produced significant treatment differences (>16%) in response rates to the Composite Lupus Assessment at Week 52 across all studies, based on the British Isles Lupus Assessment Group-based Composite Lupus Assessment (BICLA) [34]. Moreover, patients on anifrolumab at week 52 showed improved responses in a number of secondary outcomes and across various clinical endpoints, including major clinical response, BICLA, modified SRI (6), and SRI (4) [34,35]. Also, a meta-analysis of three RCTs involving 459 patients, and 468 controls confirmed that anifrolumab, as opposed to placebo, significantly increased BICLA responses. When compared to a placebo, anifrolumab also significantly decreased the use of steroids and the Cutaneous Lupus Erythematosus Disease Area and Severity Index (CLASI) scores. Moreover, anifrolumab increased SRI (7) and SRI (8) responses compared to placebo [36]. 

During the MUSE trial, an analysis of the exposure-response relationship, pharmacokinetics, and SRI (4) efficacy data indicated that intravenous injections of anifrolumab 300 mg every four weeks were the optimal dosage [37]. The entirety of the anifrolumab pharmacokinetics and pharmacodynamics data combined with the low immunogenicity has demonstrated that, overall, the approved anifrolumab 300 mg IV every four weeks regimen provides sufficient drug exposure to maximize benefit, while maintaining a tolerable safety profile in SLE patients who are receiving standard therapy [38-40].

In patients with moderate-to-severe SLE who completed the MUSE Phase IIb RCT, anifrolumab was investigated for its long-term safety and tolerability over a study period spanning three years. AEs were monitored monthly, and exploratory endpoints included disease activity, damage index, pharmacodynamics, and health-related quality of life (HRQoL) [34-36,41]. Of the patients who completed the RCT, 88.6% enrolled in the open-label extension study, with 63.8% completing the full three-year treatment. In the first year of open-label extension treatment, about 69.7% of patients reported having at least one AE, and throughout the course of three years, constant patterns of significant AEs were noted [41]. As a result, a small percentage of patients (6.9%) stopped their therapy, and no new safety flags were noted. In the IFN-high population, there was a persistent decrease in disease activity and retention of IFN-I gene signature neutralization. Overall, the safety profile of long-term anifrolumab treatment was found to be acceptable, and serologic markers, HRQoL, and SLE disease activity all showed consistent improvements [41]. 

Moreover, because patients might find subcutaneous administration more convenient than intravenous delivery, researchers sought to assess the pharmacokinetics, pharmacodynamics, safety, and efficacy of subcutaneous anifrolumab on individuals with SLE, active skin disease, and a high IFN-I gene signature. Indeed, the safety profile of subcutaneously administered anifrolumab every two weeks to SLE patients with moderate-to-severe skin manifestations remained consistent with previous studies of intravenously administered anifrolumab. This supports the ongoing development of subcutaneous anifrolumab therapy for patients with SLE [42].

Global Phase III clinical trials (TULIP 1 and 2) further evaluated anifrolumab's efficacy [43,44]. TULIP 1 (NCT02547922) and TULIP 2 (NCT02446899) were 52-week randomized, placebo-controlled trials that involved the intravenous administration of anifrolumab every four weeks. In TULIP 1, anifrolumab and placebo groups did not exhibit statistically significant differences in primary endpoints, specifically in BICLA response. On the other hand, TULIP 2 demonstrated noteworthy advancements in various parameters, such as the BICLA response rate at week 52, irrespective of the presence of the IFN signature. Furthermore, TULIP 2 showed noteworthy advancements in important secondary outcomes including the reduction in glucocorticoid dose to ≤7.5 mg/day, sustained from week 40 to week 52, a reduction of ≥50% in the Cutaneous Lupus Erythematosus Disease Area and Severity Index (CLASI) at week 12, a reduction of ≥50% from baseline in counts of swollen and tender joints at week 52, and the annualized flare rate through week 52 [43,44]. 

Overall, unlike previous trials with rontalizumab and sifalimumab, anifrolumab demonstrated efficacy in tested groups, with statistically significant effects on secondary endpoints. Ultimately, these findings suggested that targeting IFN-I in SLE patients is associated with clinical efficacy and safety, which ultimately supports the approval of this drug by several regulatory agencies [45]. In particular, in a critical appraisal of evidence that led to FDA approval of anifrolumab for SLE treatment, it has been stated that anifrolumab was found to be effective in lowering disease activity and improving clinical outcomes in patients with moderate-to-severe SLE, although the occurrence of certain AEs warrants attention [46]. In particular, with the exception of a slightly higher incidence of some viral infections, such as varicella zoster and herpes zoster, as well as possibly influenza, nasopharyngitis, and bronchitis, especially in patients without specific comorbidities or chronic infections, AEs were generally comparable between the anifrolumab and placebo groups [34,36,41,43,47-51]. This may prompt specific precautions such as preemptive vaccination and individual risk-benefit assessments.

Assessing Pharmacokinetics, Safety, and Efficacy of Anifrolumab in Various Patient Subgroups With Moderate-to-Severe SLE

Moreover, pooled data from two phase III trials were analyzed to characterize the relationship between anifrolumab pharmacokinetics, efficacy, and safety in patients with moderate to severe SLE despite standard therapy [38]. The analysis included patients receiving anifrolumab (150 mg or 300 mg) or placebo, with a focus on those who completed treatment and had high interferon gene signature (IFNGS). BICLA and SRI (4) response rates were evaluated at week 52 using exposure-response analysis in relation to the average serum concentration of anifrolumab (Cave). Cave was found to be a significant covariate in the anticipated BICLA response by logistic regression, meaning that higher anifrolumab Cave was associated with increased efficacy. Anifrolumab 300 mg was preferred over placebo in BICLA and SRI (4) treatment differences across all Cave subgroups and analytic populations. Importantly, neither anifrolumab dose was associated with exposure-driven safety events in any of the individuals [38]. 

Another pooled analysis of phase II and III trials was carried out to evaluate the safety and tolerability of anifrolumab in adults with moderate to severe SLE. According to the study, anifrolumab was generally well tolerated during a 52-week period, with AEs occurring in 86.9% of participants as opposed to 79.4% in the placebo group [48]. 

Moreover, a long-term extension study investigated the safety of anifrolumab 300 mg compared to placebo in SLE patients who completed the TULIP phase III trial [52]. Patients continued anifrolumab 300 mg or switched from anifrolumab 150 mg to 300 mg or were re-randomized from placebo to either anifrolumab 300 mg or placebo every four weeks. Results showed that SAEs and AEs leading to treatment discontinuation were lower with anifrolumab compared to placebo. Moreover, COVID-related AEs were higher with anifrolumab, but no COVID-related AEs occurred in fully vaccinated individuals. Malignancy and major cardiovascular events were low and comparable between groups. Anifrolumab was associated with lower glucocorticoid use and greater improvement in the SLE Disease Activity Index 2000 (SLEDAI‐2K). Overall, the study supported the favorable long-term benefit-risk profile of anifrolumab for patients with moderate-to-severe SLE [52].

Anifrolumab's safety and effectiveness in treating SLE were investigated in another post hoc analysis using pooled data from the phase III TULIP 1 and TULIP 2 studies, taking into account differences in patient subgroups. These subgroups included those based on serological markers, clinical features, demographic parameters, and IFNGS. When compared to IFNGS-low individuals, IFNGS-high patients typically had higher baseline disease activity and aberrant serological markers. Subgroups showing larger treatment differences included IFNGS-high patients, those with abnormal baseline serological markers, and Asian patients [53]. This brings attention to potential variations across different subgroups and emphasizes how crucial it is to carefully choose patients in order to maximize treatment outcomes [54-57].

In particular, in another post hoc analysis of the phase III TULIP-2 trial, the efficacy and safety of anifrolumab in Japanese patients with SLE were evaluated [54,56]. In this study, 362 patients on conventional care were randomized to receive 300 mg IV anifrolumab every four weeks or a placebo during a 52-week period. Anifrolumab's safety and tolerability profile in SLE patients from Japan were in line with what was shown in the TULIP-2 cohort as a whole. According to these results, anifrolumab 300 mg is safe and efficacious for SLE patients in Japan, which is consistent with its established clinical profile in the larger trial group [54,56]. Overall these studies suggest consistent efficacy and safety of anifrolumab across a range of patients with moderate-to-severe SLE [53-56].

In order to provide a comprehensive overview of the efficacy and safety of anifrolumab in the management of moderate-to-severe SLE, a table summarizing the key findings from the mentioned clinical trials was compiled (Table 2).

**Table 2:** Key findings from clinical trials evaluating the safety and effectiveness of anifrolumab in moderate-to-severe SLE. AEs, adverse events; LTE, long-term extension; SAEs, serious adverse events; RCT, randomized controlled trial; SLE, systemic lupus erythematosus

Study Title	Study Design	Patient Population	Intervention	Primary Endpoint(s) Achieved	Secondary Endpoint(s) Achieve	AEs	
MUSE phase IIb [34,35,37,39-41]	RCT	Adults with moderate-to-severe SLE	IV anifrolumab (300 mg or 1,000 mg) or placebo every 4 weeks	SLE Responder Index SRI (4) response at week 24	Composite Lupus Assessment at Week 52, major clinical response, modified SRI (6), and SRI (4) at week 52	Higher incidence of viral infections (varicella zoster, herpes zoster, possibly influenza, nasopharyngitis, and bronchitis); 69.7% reported at least one AE in first year; 6.9% discontinued due to AEs; no new safety flags	
Global phase III trials (TULIP 1 and TULIP 2) [34,36,43,44,46-51]	RCT	Patients with moderate-to-severe SLE	IV anifrolumab (300 mg) every 4 weeks	Significant improvements in BICLA response rate at week 52 (TULIP 2)	Reduction in glucocorticoid dose to ≤7.5 mg/day, from Week 40 to 52; reduction of ≥50% in CLASI at Week 12; reduction of ≥50% in counts of swollen and tender joints at Week 52; annualized flare rate through Week 52	Higher incidence of viral infections (varicella zoster, herpes zoster, possibly influenza, nasopharyngitis, and bronchitis); AEs generally comparable to placebo	
Long-term safety assessment after MUSE phase IIb [34-36,41]	Observational	SLE patients completing MUSE phase IIb RCT	IV anifrolumab (300 mg) every 4 weeks	Acceptable safety profile, persistent decrease in disease activity, improvement in HRQoL	N/A	69.7% reported at least one AE in the first year; 6.9% discontinued due to AEs; no new safety flags noted	
LTE study after TULIP trials [52]	RCT	SLE patients completing TULIP Phase III trials	Anifrolumab 300 mg or 150 mg or placebo every 4 weeks	Lower incidence of SAEs and AEs leading to treatment discontinuation with anifrolumab compared to placebo	Lower glucocorticoid use and greater improvement in the SLEDAI‐2K	Higher incidence of COVID-related AEs with anifrolumab, but none in fully vaccinated individuals; low and comparable rates of malignancy and major cardiovascular events between groups	
Pooled analysis of TULIP 1 and TULIP 2 trials [38,48]	Observational	Adults with moderate-to-severe SLE	IV anifrolumab every 4 weeks	Safety and effectiveness consistent with trial cohorts	N/A	AEs in 86.9% of anifrolumab group vs. 79.4% of placebo group; higher incidence of certain viral infections (varicella zoster, herpes zoster, nasopharyngitis, and bronchitis)	
Analysis in Japanese patients (TULIP 2 trial) (53-57)	RCT	Japanese patients with SLE	IV anifrolumab (300 mg) every 4 weeks	Safety and efficacy consistent with larger trial group	N/A	AEs generally consistent with larger trial population; higher incidence of viral infections (varicella zoster and herpes zoster)	

Now that the drug is being widely used in the real world, it will generate the data required to understand more about the types of patients who respond best to it and when anifrolumab should be utilized in a treatment plan [58].

Recently, anifrolumab's safety and effectiveness were assessed in a retrospective observational study in SLE patients classified by Lupus Low Disease Activity State (LLDAS) in actual clinical settings (Table 3) [59]. The primary endpoint was the retention rate of anifrolumab over 26 periods following commencement. At week 12, LLDAS achievement rates were evaluated by contrasting patients who experienced mild flares with those who did not achieve LLDAS. Using propensity score adjustment, safety and effectiveness were compared with standard of care (SoC) groups receiving immunosuppressive therapy or glucocorticoids. At week 26, the retention rate for anifrolumab was 89.7%. Week 12 LLDAS accomplishment rates were 42.9% in the non-LLDAS achievement group and 66.7% in the minor flare group. The anifrolumab group, especially those with mild flares, presented considerably lower glucocorticoids dosages without significantly differing in LLDAS accomplishment compared to SoC groups. These results imply that anifrolumab may be able to successfully decrease disease activity and lower glucocorticoids dosages, particularly in patients who are having mild flare-ups following LLDAS [59].* *

**Table 3:** Findings from a retrospective observational study in clinical settings evaluating safety and effectiveness of anifrolumab in moderate-to-severe SLE. LLDAS, Lupus Low Disease Activity State; AEs, adverse events; SLE, systemic lupus erythematosus; SoC, standard of care

Study Title	Patient Population	Intervention	Primary Endpoint(s) Achieved	Secondary Endpoint(s) Achieved	AEs
Retrospective observational study in clinical settings [58,59]	SLE patients classified by LLDAS	IV anifrolumab	Retention rate of anifrolumab over 26 weeks, LLDAS achievement rates at week 12, safety comparison with SoC groups	Lower glucocorticoids dosages in anifrolumab group; no significant differences in LLDAS accomplishment compared to SoC groups	N/A

Discussion 

The results of this systematic review shed important light on the long-term effects, safety, and efficacy of anifrolumab in the management of moderate-to-severe SLE. Following a thorough assessment in several clinical trials such as the MUSE Phase II and the TULIP Phase III trials, anifrolumab showed notable effectiveness in meeting key endpoints, including major clinical response, BICLA, CLASI, and SRI response [34-41,43,44]. Patients treated with anifrolumab demonstrated significant improvements in skin disease, a sustained reduction in oral corticosteroids, and a lower frequency of flare-ups when compared to those receiving a placebo [34]. Furthermore, anifrolumab's long-term safety and tolerability were evaluated over the course of a three-year study, and the findings demonstrated an acceptable safety profile with consistent improvements in disease activity, serologic markers, and HRQoL [34-36,41]. Notably, side effects were mostly under control, and only a small number of patients had a slightly elevated risk of some viral infections, including varicella-zoster and herpes zoster, as well as possible influenza, nasopharyngitis, and bronchitis [34-36,47,49-51].

It was shown that anifrolumab's pharmacokinetics and pharmacodynamics were most effective in optimizing therapeutic effects while keeping a manageable safety profile. This was especially true when the drug was administered intravenously at a dose of 300 mg every four weeks, even in a long-term assessment study [37,52]. Moreover, evidence showing similar safety and efficacy to intravenous administration also backed the convenience of anifrolumab subcutaneous dosing every two weeks, which would ultimately enhance patient convenience and comfort while maintaining therapeutic efficacy and safety [42].

Asian patients and those with a strong IFNGS were among the patient groups for which subgroup analysis demonstrated anifrolumab's effectiveness [53-57]. In these subgroups, administering anifrolumab has demonstrated steady efficacy and safety, which supports its validation as a viable SLE therapy choice by regulatory agencies [32,33]. These results highlight the significance of personalizing treatment plans to the unique features of each patient since they imply that specific biological and demographic variables may affect how well a medication works. Anifrolumab's application in the real world is expected to provide priceless insights into the subtleties of patient response patterns as data mounts. By means of extensive observation and examination of results across many clinical contexts, scientists and medical professionals can gain a more profound comprehension of the patient characteristics that are most advantageous for positive reactions to anifrolumab treatment. With the support of this increasing amount of data, anifrolumab can be optimally incorporated into individualized treatment plans for patients with SLE, allowing for more complex and personalized approaches to treatment decision-making.

The results of this systematic analysis are reinforced by empirical data demonstrating anifrolumab's efficacy and retention rate in patients classified according to LLDAS [59]. According to these results, anifrolumab therapy may be useful in lessening the severity of SLE and, consequently, the requirement for glucocorticoid medication, especially in individuals who experience mild flare-ups after reaching LLDAS. These findings point to a possible direction for tailored therapeutic approaches meant to maximize disease control and reduce side effects associated with treatment. This suggests the importance of taking into account unique patient features and disease trajectories when making treatment decisions, highlighting the potential of anifrolumab to meet the unique needs of patients navigating the challenges associated with managing SLE.

Moreover, given the proven efficacy and safety of anifrolumab in treating SLE, there has been a growing interest in exploring additional treatment avenues for this drug. For instance, it has been suggested that further drug development efforts targeting plasmacytoid dendritic cells and TLR may provide additional benefits given their role in interferon production [45]. By targeting these components, one may strategically intervene early in the immune response cascade and maybe lessen the hyperactive immunological response that is a hallmark of SLE. By modulating the activity of plasmacytoid dendritic cells and TLR, it may be possible to more effectively regulate interferon production, thereby exerting a broader influence on the immune dysregulation underlying SLE pathology. When combined with anifrolumab, this multimodal approach may have synergistic benefits that improve clinical outcomes and help better manage the condition in SLE patients.

In addition, a study evaluated the impact of anifrolumab on neutrophil dysregulation and cardiometabolic disease markers in patients with moderate-to-severe SLE. Specifically, it was demonstrated that anifrolumab markedly decreased the production of NETs, tumor necrosis factor (TNF), and interleukin-10 (IL-10) levels, all of which were correlated with IFN-I pathway activity. Anifrolumab treatment improved dysregulated cardiometabolic disease markers, such as glycoprotein acetylation (GlycA) and cholesterol efflux capacity (CEC), relative to baseline. These results imply that anifrolumab, which inhibits the IFN-I pathway, may reduce the risk of cardiovascular disease in SLE patients by modifying the variables that lead to SLE vasculopathy [60].

Another recent study demonstrated that anifrolumab holds promise in addressing refractory cutaneous manifestations in patients with cutaneous lupus erythematosus (CLE), including those with SLE. Its effectiveness in alleviating cutaneous symptoms is supported by evidence from the literature and clinical instances, despite the paucity of real-world efficacy and safety data. Specifically, it was reported that anifrolumab treatment produced positive results for four patients with SLE and refractory CLE. This finding suggests that patients with skin-limited lupus may also benefit from anifrolumab treatment [61].

Moreover, although not the focus of this systematic review, anifrolumab is being evaluated in lupus nephritis, one of the most common severe organ manifestations of SLE, occurring in up to ∼50% of patients [62,63]. In particular, the safety and tolerability profile in the TULIP-LN trial (NCT02547922) has been shown to be generally acceptable, with promising efficacy results for the patients receiving an intensified regimen of 900 mg for the first three doses followed by 300 mg anifrolumab. Collectively, the results support further investigation of an anifrolumab-intensified dosing regimen in larger populations of patients with active proliferative lupus nephritis.

## Conclusions

Overall, this systematic review offers a thorough understanding of the effectiveness, safety, and long-term consequences of anifrolumab in the management of moderate-to-severe SLE. To assess these points, the results obtained from the MUSE Phase II and TULIP Phase III trials have been evaluated. The analysis resulted in the finding that anifrolumab significantly improved patient outcomes, while remaining safe to use and generally well tolerated. Also, across prolonged trial periods, it was shown that the use of this drug resulted in sustained improvements in disease activity and quality of life for affected patients, ultimately indicating that its long-term safety profile is still positive. Moreover, anifrolumab's efficacy in treating particular patient groups, such as Asian patients and those with a strong interferon gene signature, has also been highlighted by subgroup studies, which adds to the evidence supporting its acceptance as a treatment option for SLE.

The increased interest in investigating alternative therapy options, such as targeting toll-like receptors and plasmacytoid dendritic cells, underlines the potential for more innovation in SLE management strategies. Additionally, there were newer data points to possible advantages of anifrolumab beyond its original indications, such as effects on markers of cardiometabolic illness, neutrophil dysregulation, and refractory cutaneous symptoms in individuals affected by CLE. Although further studies are required to completely understand the wider therapeutic implications of anifrolumab, especially in lupus nephritis, these findings support further research into anifrolumab's role in personalized treatment plans for SLE.
